# Urban safety and psychological distress during the pandemic: the results of a longitudinal study

**DOI:** 10.3389/fpsyg.2024.1343585

**Published:** 2024-05-06

**Authors:** Fabio Ferretti, Giacomo Gualtieri, Alessandra Masti, Allison Uvelli

**Affiliations:** Department of Medical Sciences, Surgery and Neurosciences, University of Siena, Siena, Italy

**Keywords:** urban safety, ego-resiliency, stress, anxiety, depression, COVID-19

## Abstract

**Introduction:**

In the last decades, a large body of literature has explored the topic of perceived safety and fear of crime in urban environments. The effects of psychological factors on such feelings have been studied, but rarely using prospective studies, and never when these factors intercept a worldwide dramatic event like the pandemic. This research aimed to analyze the variations of the feelings of urban safety during the pandemic, the role of resiliency and the effect of psychological stressors such as anxiety, stress, and depression.

**Methods:**

During 2019 and 2022, before and after the pandemic, a face-to-face interview was administered to the same group of 195 participants. The PUSAS scale was used to measure unsafety, the ER89-R to assess for resiliency, the DASS-21 to collect data about the general distress (anxiety, stress and depression), and the CAS scale was used to evaluate the specific coronavirus anxiety. Structural equation models were applied to test a theoretical framework grounded on the relationships between these measures.

**Results:**

The research findings showed decreased feelings of unsafety across the pandemic, consistent with the literature. The positive effect of ego-resiliency was significant but only for its interaction with data collected before the pandemic, whilst stress and anxiety impacted unsafety in 2022 through different pathways. None of the symptoms of general distress influenced the concern about crime and sense of vulnerability, as the feelings of unsafety were found independent from the variations of the specific coronavirus anxiety.

**Discussion:**

Although the research findings did not confirm the impact of coronavirus, they presented some facets that disconfirm what the literature reported about the relationships between psychological distress and fear of crime. Implications about measurement issues are discussed.

## Introduction

Since the beginning of the ‘60s, feelings of unsafety in an urban environment have been considered one of the topics that most influenced the local and national governments’ agenda, not only because of its relevance in crime control but also because fear of being victimized negatively affects the quality of life (Alfaro-Beracoechea et al., 2018). Several studies reported a significant association between fear of crime and well-being outcomes at the individual level (Stafford et al., 2007; Jackson and Stafford, 2009; OECD, 2011; Lorenc et al., 2012), mainly concerning mental health. The review by Lorenc et al. (2012) highlighted the importance of environmental factors in mediating the relationship between fear of crime and well-being.

Few or nothing is known in the literature about the impact on the feelings of unsafety of the most important event of the last years: the pandemic, an unexpected situation that changed for long time the lives of billions of people, their behaviors, the way they lived the relationships in their community. The results of a meta-analysis by Jin et al. (2021), indicated that COVID-19 mass quarantine had important psychological impacts on individual anxiety, depression, and stress, the same psychological distresses that were frequently cited in the criminological and sociological literature on fear of crime (Wallace, 2012). Anxiety in particular was a key concept used by the scholars to describe such feelings: at the beginning of the ‘80s, Garofalo (1981) proposed a conceptualization of fear defining it as the anxiety resulting from the perception of a danger or a threat of physical harm. But other topics, such as resiliency, shared their importance in the literature concerning the psychological impacts of the pandemic and the research on fear of crime. Considering the psychological impact of the pandemic, ego-resiliency was found a protective factor against the development of anxiety and aggression (Dębski et al., 2021), or functioned to cope with the specific stresses associated with COVID-19 (Kubo et al., 2021). The literature on fear of crime also emphasized the positive effects of ego resiliency on the feelings of unsafety, pointing out that it can influence the sense of personal control and desensitize against perceived risk, operating alongside cumulative adversity (Shippee, 2012). Dutton and Greene (2010) explained the multi-faceted characteristics of resilience as protective factors related to personal traits or social and cultural factors, as a process of adaptation, or as positive outcomes following exposure to adverse events.

Many authors have tried to explain the complexity of a multidimensional phenomenon, as is the fear of crime. The latest interpretative models offered by the literature (Lorenc et al., 2014) fully capture these interrelationships’ complexity levels. They proposed a causal map which resumed six key concepts that synthesized years of debates about the definition of fear of crime and its determining factors: (1) crime and disorder (violent or potentially violent crimes against the person, drug- and alcohol-related crimes), ‘environmental’ crimes such as criminal damage, vandalism and graffiti; (2) fear of crime (perceived risk, emotional responses, individual attitudes, perceived vulnerability); (3) health and well-being (physical activity, social well-being, interpersonal interaction and social capital); (4) built environment (design of public spaces, architecture and design of residential housing, …); (5) social environment (socioeconomic status, ethnicity, structural inequalities and individual discrimination, social cohesion or integration); (6) national policies (economy, crime and justice, …).

With such a complex framework, many of these factors had probably been affected by the pandemic’s negative impact on the feelings of unsafety.

But Lorenc’s model also underlined the difficulties in defining the fear of crime. After decades of debates on this topic, there is no universal definition of fear of crime within the established literature, and its meaning varies so substantially in the literature that its measurement is in danger of losing any specificity. As currently accepted by scholars, fear of crime is a multidimensional construct conceptualized and operationalized with increasingly complex models, allowing scholars to measure the different factors contributing to the fear of crime or using fear as the dependent variable. Even the semantic representation of these feelings of worry contributed to the misrepresentation of this phenomenon: sometimes fear of crime was used interchangeably with unsafety; however, there is no clear discrimination between the two concepts (fear of crime and unsafety). From a conceptual point of view and considering the many theoretical approaches analyzed in this work, the latter should represent a broader construct, which includes the concern for crime (Ferretti et al., 2023). Clearing the ambiguity deriving from using terms such as fear of crime and unsafety as synonyms would be helpful to a more explicit definition of these phenomena.

In this research was used the definition given by Amerio and Roccato (2007) to explain the construct of unsafety. They stated that these feelings are the confluence of perceptions, judgments, feelings, emotions and concerns that emerge from the individual’s material, social and symbolic environment, a mixture of emotional and cognitive states. The individual’s perception of safety/unsafety is rooted in the characteristics of the ecological and social relationships rather than ruled by the objective assessment of the criminal risk due to the environment. A definition that also explain the discrepancy between the perception of unsafety and the actual levels of crime since unsafety is not to be attributed to the actual fear of being victimized (in many urban contexts, a rather improbable event) but to the signals that come from the surrounding environment and that create a feeling of unease in individuals, perhaps only because these signals do not correspond to values and traditions accepted in the community.

### Hypothesized model: psychological features and influences

Psychological variables, such as ego-resiliency (ER), may be expected to influence urban safety. High-resiliency people can adapt better to changing circumstances, attain higher levels, and mold to their environment (Block and Block, 1980). ER incorporates individual abilities, including ego-control, self-efficacy, and collective efficacy, which are protective factors for psychological health (Hu et al., 2015; Luthar, 2015).

Covid-era studies demonstrated that ER positively predicted personal resource gains, decreasing stress levels (Sanecka et al., 2023). ER, in its Optimal Regulation component, correlates negatively with anxiety severity (Dębski et al., 2021; Skalski et al., 2021; Florek et al., 2023). Study of Goryczka et al. (2022) also exhibited a negative link between levels of ER, and depressive and anxiety symptoms, and a positive correlation with life satisfaction during the pandemic.

Urban safety (US) is a construct that affects people’s quality of life, health, and well-being, and causes mental health ailments (Jackson and Gray, 2010; Rubijsbroek et al., 2015). A study of Macassa et al. (2023) found a statistically significant association between fear of crime and anxiety, in fact, people with high levels of fear of crime have an OR of 3.17 to have anxiety compared to people without fear of crime. Cognitive mechanisms of anxiety, stress, and depression such as cognitive triad, hopelessness, and catastrophization (Beck et al., 1979) could negatively affect the perception of safety.

Furthermore, several studies have illustrated the impact of the pandemic on child and adult mental health (Francourt et al., 2020; Racine et al., 2020). The most commonly reported symptoms included depression, anxiety, and distress whose level depends on the interplay of individual psychological resources and risk factors (Pedrosa et al., 2020; Salari et al., 2020; Panchal et al., 2021). Ahorsu et al. (2020) found a positive correlation between depression, anxiety, and fear of covid, assessing the fear of COVID-19 scale.

Worrying about catching COVID-19 has been associated with negative outcomes such as poor mental health (Sloan et al., 2020), and it may also be related to reluctance to re-engage with social activities as lockdown eases (Shaw et al., 2020). Worry can damage people’s mental health and quality of life and stimulate care and precaution. Thanks to the fear of crime studies, worry can be divided into two different categories regarding the utilized coping strategies: “functional fear,” in which people use adaptive emotions and preventive activities to help guard themselves against the cause of their worry, and “dysfunctional fear,” which involves people worrying about something and reporting that their quality of life is negatively affected by this worry and/or their precautionary behavior (Jackson and Gray, 2010). According to Solymosi et al. (2015), dysfunctional worry was associated with negative emotional outcomes that may affect people’s mental health, and functional worry did not have the same association with these negative outcomes. Authors show that, for some people, worry can be beneficial, and does not damage well-being.

Fear of crime and fear of COVID have a lot in common: adverse emotional effects upon people, inducing a feeling of isolation and vulnerability and general loss in personal well-being (Hale, 1996), motivating people to remain indoors more than they would wish or to avoid certain places (Moore and Trojanowicz, 1988); in extreme cases, it can be destructive and paralyzing.

A large body of literature explored the relationships between fear of crime and psychological factors such as general distress or resiliency, but, according to our knowledge, rarely have the authors chosen a broad definition of unsafety, like that proposed by Amerio and Roccato, to describe the feelings of worry in an urban environment. Furthermore, papers analyzing unsafety and psychological factors in a longitudinal design are even more challenging to find, but data with these characteristics explaining this relationship during the pandemic are not disposable in the literature.

This study aims to analyze the variations of perceived urban safety across the pandemic and the effects of psychological determinants, such as resiliency and general distress (anxiety, stress and depression), on the feelings of unsafety. The research, on the one hand, aims to test the hypothesis that COVID-related general distress may produce adverse effects on the variations of perceived safety and that, on the other hand, ego-resiliency may represent a protecting factor able to reduce the concerns about unsafety.

We hypothesize that a complex model is needed to explain the relationships between these different factors. The key factor could be ER. ER is a characteristic of an individual that influences the onset of psychopathology and can affect whether someone is worried or not about contracting COVID-19, as well as how safe or unsafe they perceive the urban context. We believe that people with high levels of ER will experience lower levels of stress, depression, anxiety, and fear of crime, with a negative correlation between these factors. We also expect that other factors could be associated. As already explained, fear of crime and fear of COVID are based on similar mechanisms, which means worrying about COVID could increase the perception of urban unsafety. Additionally, worrying about COVID could lead to stress, depression, and anxiety which could have a bidirectional relationship. This means that the more afraid a person is of COVID, the more anxious, depressed, and stressed they will be. Conversely, the more anxious, stressed, and depressed a person is, the more vulnerable they are to being afraid of COVID. Finally, the cognitive aspect of stress, depression, and anxiety could in turn influence the perception of urban safety.

## Materials and methods

This longitudinal observational study has been realized in two waves of interviews: the first during the summer of 2019 and the second during the same period in 2022. Data were collected in the Municipality of Grosseto, a city with approximately 80,000 inhabitants in southern Tuscany (Italy). The study was part of a broader collaboration started in 2018 between the local government and the University of Siena about the topic of urban safety; it complies with the Declaration of Helsinki and was approved by the University of Siena CAREUS (Committee for the ethical research in human and social sciences). All the subjects enrolled in the research signed an informed consent with full information about the study and its aims. In the beginning, the study intended to analyze the role of ego-resiliency in the variations of perceived urban safety using a longitudinal design, but the pandemic unexpected event forced the research group to introduce other measures to take into consideration the psychological impact of the long periods of mass quarantine and the subsequent restrictions to the daily routines. Being at least 25 years old, residing in the Municipality of Grosseto, speaking Italian, and being available for future interviews were considered inclusion criteria. Changing the address between the two waves of interviews was the only exclusion criterion. Only questionnaires that have been fully completed were taken into consideration.

### Participants

In 2019, a random sample was selected from the population of the Municipality’s citizens over 18 years old, with proportional assignment according to the population density of five urban areas, which were used to divide the city into neighborhoods with homogeneous characteristics. The sample size for the first wave of interviews was set at 700 participants for an estimation accuracy of 2.5% with a probability of 95%. During the summer of 2019, before the beginning of the COVID-19 pandemic, a group of trained volunteers administered 726 face-to-face interviews. After completing the questionnaire, the participants were asked if they wanted to participate in the second wave of data collection. One hundred ninety-five citizens consented to be contacted for involvement in the project’s second phase. This subsample participated in the second wave of interviews during the summer of 2022, just after the pandemic. Their address was verified to check for people moving from their homes between 2019 and 2022. Data were collected by administering the questionnaires again with the face-to-face technique. Considering the feeling of unsafety and the resiliency trait, this subsample seemed to belong to the same population describing the subjects who refused to participate in the project’s second phase. The Mann–Whitney-*U* test was used to analyze the differences in the Ego-Resiliency and PUSAS scores between the two groups of participants, and no significant differences were detected. Subjects who consented to participate in the second wave of interviews showed a mean age significantly higher than those who refused this collaboration (respectively 57.7 ± 16.0 and 52.8 ± 19.5; *p* ≤ 0.002).

### Measures

In 2019, the tools foreseen for the data collection aimed to analyze the perception of urban safety and the ego resiliency trait, respectively assessed through the PUSAS and ER89-R scales. The PUSAS scale was used again in the second wave of interviews (summer 2022), and measures of coronavirus anxiety and general distress were added, collecting these data through the CAS and DASS-21 scales. All these scales are briefly described in the following sections.

### Perceived urban safety

Perceived urban safety was measured in both interview waves by the PUSAS scale (Perceived Urban Safety Assessment Scale) (Ferretti et al., 2019), a 27-item scale which describes the theoretical construct of perceived urban safety through the following dimensions:

- Physical and social disorder (PSD; 10 items): it covers the individual’s perception of the environmental conditions of the neighborhood; vacant or abandoned housing, vandalized and run-down buildings, abandoned cars, graffiti, and litter in the streets can exemplify physical disorder (Kelling and Wilson, 1982; Skogan, 1990; Sampson and Raudenbush, 1999), whilst social disorder is related to behaviors in public places such as people drunk or taking drugs on the streets, drug dealing, hostile arguing, conflict and fighting, people loitering, rowdy groups and gang activity, street prostitution.
- Collective efficacy (CE; 9 items): it is grounded on the measurement of collective efficacy according to the paradigm proposed by Sampson and Raudenbush (1999); the items describe the quality of social relations in terms of neighborhood cohesion and the capability of exerting informal social control.
- Concerns about crime and sense of vulnerability (CCSV; 8 items): this dimension is specified according to the theoretical approaches that Killias (1990) and Gabriel and Greve (2003) proposed for concerns about crime and the sense of vulnerability. The first describes the cognitive aspects of fear and the resulting behavioral issues, such as the consequences on habits and lifestyle due to concerns about crime; the second is related to the risk perception and sense of lack of control over the severity of the outcomes of a crime.

The score of each dimension is provided by the sum of the values of the 5-point Likert scale used for the items, stating the participant’s degree of agreement (1: totally false to me; 5: totally true to me). PSD scores range from 10 to 50, CE from 9 to 45, and CCSV from 8 to 40. High scores in PSD and CCSV are related to high levels of unsafety, whilst high scores on the EC dimension correspond with lower levels of unsafety. The PUSAS’ total score is given by the sum of the 27 items, reversing those of the dimension CE; high scores on the PUSAS scale suggest high levels of perceived unsafety.

### Ego resiliency

The ego resiliency assessment was conducted during the first wave of interviews (2019) with the Italian version of the ER89-R (Alessandri et al., 2007). It is a self-report brief inventory of ego-resiliency in late adolescents and adults composed by 10 items. The construct of ego resiliency is explained through two dimensions, Optimal Regulation (OR, 6 items) and Openness to Life Experienced (OL, 4 items) which were rated on a seven-point scale, which ranges from 1 (never) to 7 (always). The OR dimension denotes agreeableness and self-regulatory abilities; OL is related to openness and curiosity. For each dimension, the total score is obtained by the sum of the items’ scores, and the final score corresponds to the sum of the two-dimension score. High scores indicate high levels of resilience.

### General distress

The Italian version of the DASS-21 (Bottesi et al., 2015) was administered to collect a measure of general distress to analyze a pattern of conditions characterizing general psychopathology and mood problems in the adult population. This self-report questionnaire provides a measure of the theoretical construct based on three distinct scales (Depression, Anxiety and Stress) for a total of 21 items, 7 for each scale, with a Likert scale response from 0 (not suitable to me at all) to 3 (very suitable to me). No reverse items are foreseen. The depression scale assesses a lack of incentive, low self-esteem, and dysphoria; the items contained in the anxiety scale refer to somatic and subjective symptoms of anxiety or acute responses of fear; and lastly, the stress scale evaluates irritability, impatience, tension, and persistent arousal. For each scale, the total score, obtained by adding the single-item score, must be multiplied by two. Scores higher than 27 in the stress scale indicate severe o extremely severe symptoms, whilst the same condition is assessed in the anxiety or depression scale when the scores are, respectively, higher than 15 and 21.

### Coronavirus anxiety

The CAS (Lee, 2020) is a self-report questionnaire composed of 5 items valuable to identify probable cases of coronavirus anxiety. It is validated on a large sample of adults and shows high reliability.

Items are grounded on the psychology of fear and anxiety literature. Each item aims to capture a unique manifestation of the particular form of Coronavirus Anxiety. These included cognitive, emotional, and physiological items. Each item was rated on a 5-point scale to reflect the symptom frequency, ranging from 0 (not at all) to 4 (nearly every day) over the preceding 2 weeks. The total score is given by adding the single-item score; no reverse items are foreseen. The pathological cut-off is 9.

### The theoretical model

**Figure 1:**
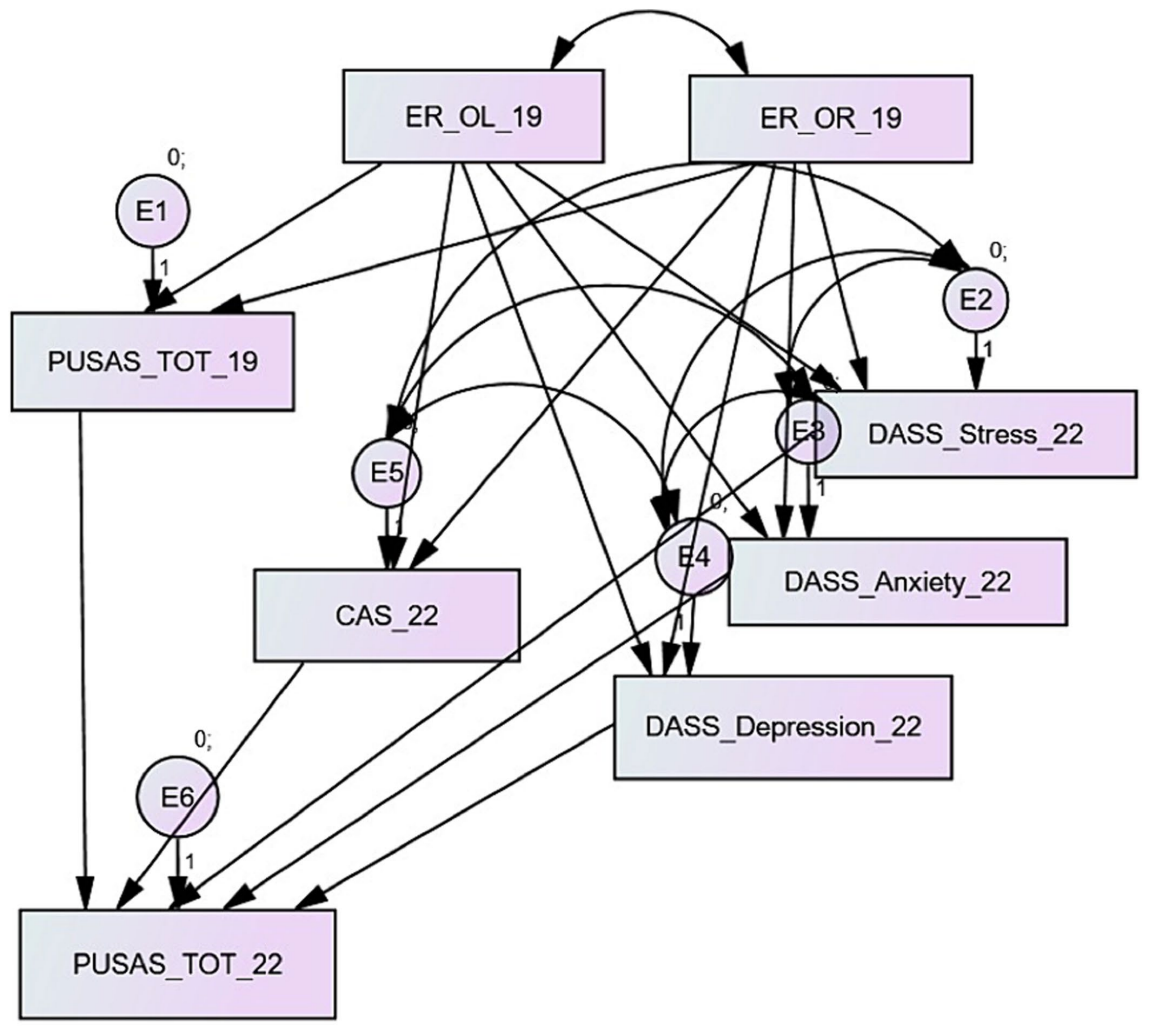
The theoretical model.

Figure 1 displays the theoretical model that this research aims to test. The network of relationships accounted for 8 exogenous and 6 endogenous variables; only the ego resiliency dimensions (openness to life experiences and optimal regulation) were considered exogenous. An error term was included in each endogenous variable.

The perceived urban unsafety measured in 2022 (PUSAS_TOT_22) is the dependent variable of the measure collected in 2019 (PUSAS_TOT_19), the coronavirus anxiety assessed in 2022 (CAS_22), and the general distress measured in 2022 (DASS S_22, DASS_A_22, DASS_D_22). The Ego resiliency evaluated in 2019 (OR_19 and OL_19) predicts the perceived urban safety measured in 2019 of the coronavirus anxiety and general distress collected in 2022.

Some covariances were introduced where necessary to explain the interdependence between variables: for example, between the two dimensions of ego resiliency or between the CAS scale and the DASS’ three dimensions. Since CAS and DASS are endogenous variables, the covariances have been set between the error terms. The model fitting showed the necessity of improvements that are described in the results section. This study tested the fitting of four models, one for each dimension of the PUSAS (physical and social disorder, collective efficacy and concerns about crime and sense of vulnerability) and for the unsafety general measure provided by the PUSAS total score.

### Statistical analysis

Descriptive statistics were used to summarize the main characteristics of the sample according to quantitative and categorical data (mean, standard deviation, median, interquartile range and frequency distributions). The Kolmogorov–Smirnov test was used to assess normality. The scores resulting from the PUSAS scale in 2019 and 2022 were compared by the non-parametric Mann–Whitney U test, while the correlations between the scores provided by CAS, DASS 21, ER and PUSAS were analyzed with the non-parametric index Spearman’s Rho.

The model fitting was analyzed by the indexes provided by the SEM analysis, starting from the value of χ^2^ (Hu and Bentler, 1999), which, however, is strongly dependent on the sample size (Jöreskog et al., 2000; Byrne, 2001). The following absolute fit indexes were considered. The CMIN/DF ratio (Wheaton et al., 1977): although there is no consensus regarding an acceptable value for this statistic, recommendations range from as high as 5.0 (Wheaton et al., 1977) to as low as 2.0 (Tabachnick and Fidell, 2007). The RMSEA (Steiger, 1990): a cut-off value close to 0.06 (Hu and Bentler, 1999) or a stringent upper limit of 0.07 (Steiger, 2007) seems to receive the consensus amongst scholars in this area. The NFI (Bentler and Bonnet, 1980): values greater than 0.90 indicating a good fit, but other authors stated that the cut-off criteria should be NFI ≥ 0.95 (Hu and Bentler, 1999; Loehlin, 2004). The RFI (Byrne, 2010): values greater than 0.95 indicate perfect fit and as with other indices values.

greater than 0.90 and above are also adequately acceptable. The CFI (Bentler, 1990): a value of CFI ≥ 0.95 is presently recognized as indicative of a good fit (Hu and Bentler, 1999; Loehlin, 2004).

The Modification Indexes were used to optimize the model. The covariance between the error terms was introduced where necessary (Bollen, 1989; Hu and Bentler, 1999). The existence of these covariances was justified by the presence of common causes of errors that affect the measurement of these indicators. The standardized regression weights were used to assess the magnitude of the relationships. Regarding the sample size used for the SEM analysis, Bentler and Chou (1987) suggested at least 5 cases for each model’s parameter, and this work met this criterion.

All analyses were performed with the statistical package SPSS -IBM v26, while AMOS package v25 was used for SEM analysis. Results are considered significant with a value of *p* < 0.05.

## Results

The mean age was a bit higher than the general population (57.7 ± 15.98) due to the selection of citizens over 18 years old. The 54.7% of the participants were females and the married marital status was found in most of the sample (74.2%), with a mean of 2.7 ± 1.13 family members.

Table 1 displays the main characteristics of the sample about the scores obtained by the tools administered to the sample. The evaluation of ego resiliency showed a total score above the range central point: the median score was 49.3 (IQR: 11.00), in a range between 10 and 70. The ego resiliency trait of the participants was represented mainly by the Optimal Regulation rather than the Openness to Life Experiences: the median value of the OR was 32.0 (IQR: 6.00; min = 6, max = 42), while the OL median was 17.7 (IQR: 6.00; min = 4, max = 28). The pathological symptoms of Coronavirus anxiety are rare in the sample examined: only 2.6% of the participants showed a CAS score higher than the pathologic cut-off (median: 0.0; IQR: 2.00). The measures concerning the general distress revealed that 7.9% of the sample suffered of severe or extremely severe symptoms of stress, 9.5% the same levels of anxiety (6.3% had extremely severe symptoms), and 9.4% severe or extremely severe symptoms of depression. Considering the median scores computed for the three dimensions of the DASS-21, stress showed the highest value (8.0; IQR: 12.00).

**Table 1:** Descriptive statistics of the measurement scales administered in 2019 and 2022 (mean and standard deviation, median and interquartile range); comparison of the PUSAS’ scores between the two waves of interviews.

	2019		2022		Statistic	*p*-value
	Mean (SD)	Median (IQR)	Mean (SD)	Median (IQR)		
**Ego resiliency**						
Optimal regulation (ER OR)	31.6 (0,33)	32.0 (6.00)				
Openness to life experiences (ER OL)	17.7 (0.36)	17.7 (6.00)				
Total score (ER TOT)	49.3 (0.52)	49.3 (11.00)				
**DASS 21**						
Stress (DASS S)			9.4 (0.67)	8.0 (12.00)		
Anxiety (DASS A)			5.0 (0.56)	2.0 (6.00)		
Depression (DASS D)			7.3 (0.65)	4.0 (10.50)		
**CAS**			1.7 (0.20)	0.0 (2.00)		
**PUSAS**						
Physical and Social Disorder (PUSAS PSD)*	23.1 (0.62)	21.0 (11.00)	21.4 (0.60)	20.0 (11.30)	M-W = –2.201	0.028
Collective Efficacy (PUSAS CE)*	32.5 (0.54)	32.5 (11.00)	31.8 (0.52)	32,0 (9.30)	M-W = –1.030	0.303
Concern about Crime and Sense of Vulnerability (PUSAS CCSV)*	22.4 (0.55)	21.0 (11.00)	18.5 (0.49)	18.0 (9.30)	M-W = –6.048	<0.001
Safety Total Score (PUSAS TOT)*	67.1 (1.14)	67.0 (20.00)	62.3 (1.13)	62.2 (21.3)	M-W = –3.337	<0.001

*Mann–Whitney *U* test (two-sided).

The data relating to perceived urban safety returned a relatively positive framework, with scores expressing low worry about the factors determining unsafety. The median of the total score was 67.0 in the first wave of interviews and 62.2 in the second (the range was between 27 and 135), the physical and social dimension scored a median of 21.0 in 2019 and 20.0 in 2022 in a range between 10 and 50, the concerns about crime and sense of vulnerability showed a median of 21.0 in 2019, while the value of 2022 was 18.0, ranging between 8 and 40. Due to the reversed scores, the collective efficacy provided a median closer to the upper limit of the range (32.5 in 2019, 32.0 in 2022, in a range between 9 and 45). The comparison of the perceived urban safety assessed in 2019 and 2022, at the beginning and after the pandemic, showed a significant decrease in the sense of unsafety regarding physical and social disorder (M-W = –2.201; *p* < 0.028), the concern about crime and sense of vulnerability (M-W = -6.048; *p* < 0.001), and the PUSAS total score (M-W = –3.337; *p* < 0.001). The decrease in collective efficacy was not significant.

The correlations between the measures are depicted in Table 2. Even with a mild intensity, the ego resiliency’s openness to life experiences dimension was significantly correlated with some dimensions of perceived safety in 2019 but not with its measurement in 2022. OL was negatively associated with physical and social disorder, collective efficacy and concern about crime. The second facet of ego resiliency (optimal regulation) showed a positive correlation with collective efficacy in 2019 and 2022 and a negative association with the PUSAS scale total score. High levels of coronavirus anxiety (CAS scale) were significantly correlated in 2019 and 2022 with high levels of unsafety in some dimensions (physical and social disorder and concern about crime) and with the total score, but not with the collective efficacy. The coronavirus anxiety was also significantly correlated with all the general distress dimensions: high scores on the CAS scale were associated with high levels of stress, anxiety and depression (both measures were collected in 2022). The degree of general distress was related to the sense of safety measures in 2022 but not in 2019. In particular, a positive correlation was found between the DASS-21 three facets and the PUSAS’ total score, the physical and social disorder, the concern about crime and the sense of vulnerability. No significant correlation was detected between stress, anxiety, depression and the collective efficacy.

**Table 2:** Spearman’s **ρ** correlation between the scores provided by the assessment tools administered in the study.

	PUSAS PSD 19	PUSAS CE 19	PUSAS CCSV 19	PUSAS TOT 19	PUSAS PSD 22	PUSAS CE 22	PUSAS CCSV 22	PUSAS TOT 22	CAS	DASS S	DASS A	DASS D
ER OR	−0.046	0.250**	−0.088	−0.166*	0.019	0.150*	−0.067	−0.065	0.017	−0.13	−0.046	−0.135
ER OL	−0.177*	−0.151*	−0.169*	−0.126	0.028	−0.048	−0.097	0.006	−0.018	0.037	0.08	0.035
ER TOT	−0.122	0.049	−0.166*	−0.180*	0.054	0.057	−0.071	−0.013	−0.008	−0.055	0.000	−0.069
PUSAS PSD 19					0.451**	−0.070	0.261**	0.395**	0.162*	0.076	0.123	0.097
PUSAS CE 19					0.007	0.379**	0.079	−0.137	−0.083	−0.066	0.016	−0.122
PUSAS CCSV 19					0.209**	0.103	0.327**	0.237**	0.204**	0.049	0.054	0.021
PUSAS TOT 19					0.313**	−0.196**	0.270**	0.389**	0.249**	0.096	0.075	0.138
PUSAS PSD 22									0.188**	0.317**	0.273**	0.275**
PUSAS CE 22									−0.025	−0.134	−0.024	−0.167*
PUSAS CCSV 22									0.222**	0.267**	0.291**	0.252**
PUSAS TOT 22									0.226**	0.349**	0.285**	0.343**
CAS										0.422**	0.397**	0.414**

*p < 0.05. **p < 0.01.

The analysis of the correlations between the dimensions of the PUSAS scale in the two waves of interviews showed that the physical and social disorder scored in 2019 was positively with the total score and the concern about crime in 2022. The same association was observed between the concern about crime in 2019 and the total score and the physical and social disorder in 2022. The total score in 2019 was positively correlated with all the dimensions of the PUSAS scale, except for the collective efficacy that provided a negative correlation, but this is coherent with the reverse score of this dimension. Looking at this last facet, data revealed that the measures collected in 2019 were only correlated with the same data in 2022, but non correlated with the other scale’s dimensions.

As described in the materials and method section, the data fitting to the theoretical model hypothesized in this study (see Figure 1) was assessed with the structural equation modeling. The analysis of the modification indexes suggested the inclusion of a covariance between the error terms of the PUSAS measurement in 2019 and the CAS administered in 2022.

Four models have been fitted, one for each PUSAS dimension and one for the scale’s total score. The following are the results of these analyses.

**Figure 2:**
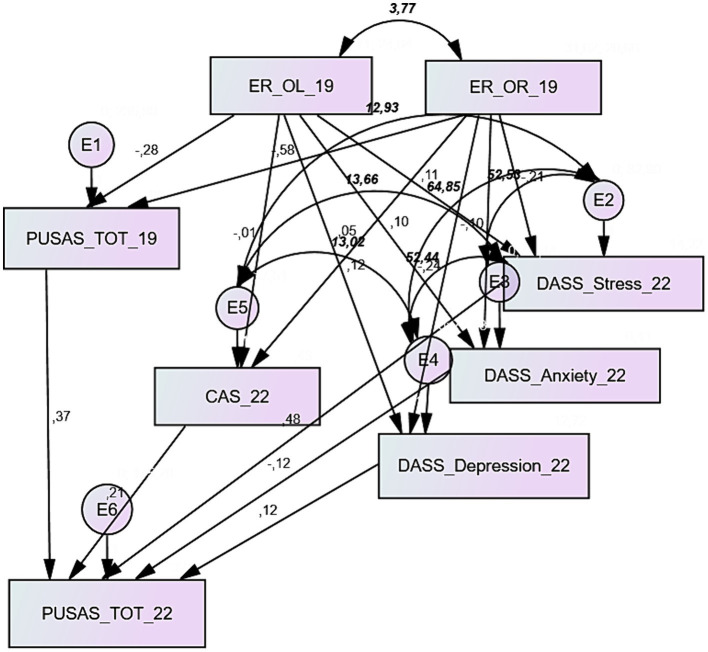
Path diagram of the model interpreting the PUSAS total score; parameters in regular text explain the standardized estimates of the regression weights, and parameters in bold italics explain covariances.

Figure 2 displays the model interpreting the PUSAS total score. Data in the path diagram expressed the standardized regression weights or the covariances in the network of relationships depicted by the graph. The model showed an excellent fit (Table 3): absolute and relative fit indexes agreed completely with the cut-off criteria cited in the literature. The CMIN/DF ratio was largely below 2.0, the value of RMSEA was 0.000, and NFI, RFI, and CFI all presented measures above 0.95.

**Table 3:** Estimate, standard error, standardized estimates, and significance of the model’s regression weights (PUSAS total score); absolute and relative indexes assessing the goodness of fit.

		Estimate	S.E.	Standardized estimate	*p-*value
ER OL ➔ PUSAS TOT 19		−0.28	0.228	−0.089	0.219
ER OL ➔ CAS		−0.01	0.040	−0.019	0.799
ER OR ➔ PUSAS TOT 19		−0.585	0.249	−0.169	**0.019**
ER OR ➔ CAS		0.046	0.044	0.078	0.292
ER OL ➔ DASS D		0.115	0.131	0.065	0.378
ER OL ➔ DASS A		0.099	0.114	0.064	0.385
ER OL ➔ DASS S		0.108	0.135	0.059	0.424
ER OR ➔ DASS D		−0.237	0.143	−0.122	0.097
ER OR ➔ DASS A		−0.099	0.125	−0.058	0.428
ER OR ➔ DASS S		−0.214	0.148	−0.106	0.147
PUSAS TOT 19 ➔ PUSAS TOT 22		0.375	0.063	0.383	**0.000**
CAS ➔ PUSAS TOT 22		0.210	0.467	0.037	0.652
DASS S ➔ PUSAS TOT 22		0.481	0.191	0.287	**0.012**
DASS A ➔ PUSAS TOT 22		−0.118	0.228	−0.059	0.605
DASS D ➔ PUSAS TOT 22		0.12	0.206	0.069	0.560
	CMIN/DF	RMSEA	NFI	RFI	CFI
Goodness of fit indexes	0.848	0.000	0.993	0.960	1.000

Bold values are significant values.

Table 3 showed also the regression weights and the standardized regression weights of the relationships described by the model. Only three of these provided a significant value: the negative regression weight of the effect of ego resiliency’s optimal regulation on the PUSAS total score in 2019 (standardized γ: −0.169; *p* < 0.019), the positive parameter of the PUSAS total score in 2019 on the PUSAS total score in 2022 (standardized γ: 0.375; *p* < 0.000) and the positive regression weight of the stress measured by the DASS-21 on the PUSAS total score in 2022 (standardized γ: 0.481; *p* < 0.012). All the model’s covariances were significant and with positive signs.

**Figure 3:**
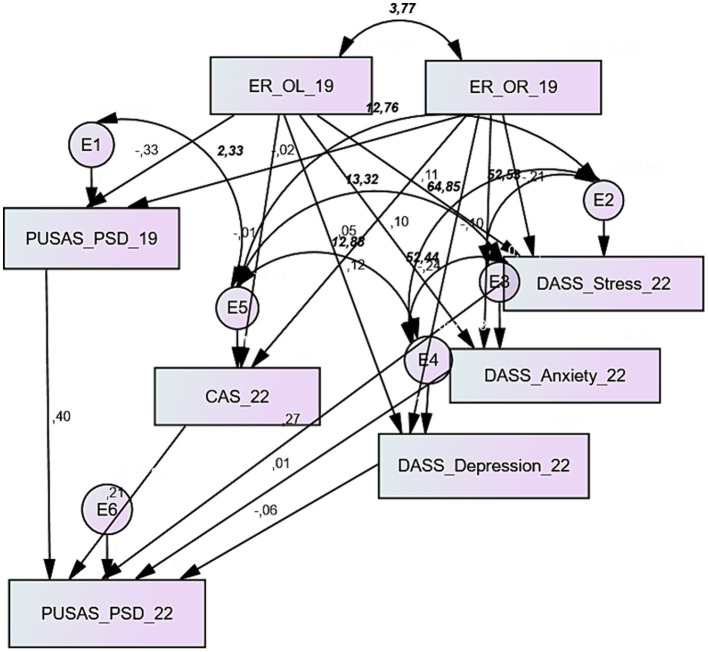
Path diagram of the model interpreting the PUSAS Physical and Social Disorder score; parameters in regular text explain the standardized estimates of the regression weights, and parameters in bold italics explain covariances.

The model’s path diagram interpreting the PUSAS Physical and Social Disorder score is depicted in Figure 3. The model fitting provided good fit indexes (Table 4): the CMIN/DF ratio was below 2.0 (1.837), RMSEA was close to 0.000 (0.067), NFI and CFI showed values above 0.95 (respectively 0.985 and 0.963), whilst RFI was 0.914, slightly below the cut-off of 0.95. The model’s significant relationships almost overlapped those provided by the PUSAS total score model: the 2019 score in physical and social disorder was a significant predictor of its measure collected in 2022 (standardized γ: 0.395; *p* < 0.000), and the effect of the stress dimension of the DASS-21 was significant as well on the PUSAS physical and social disorder score in 2022 (standardized γ: 0.273; *p* < 0.006), both with positive standardized regression weights. The effect of ego resiliency on the physical and social disorder was significant, with a negative regression weight, for the relationships with openness to life experiences (standardized γ: −0.334; *p* < 0.007), and not with the optimal regulation, as the PUSAS total scores’ model. All the covariances were significant.

**Table 4:** Estimate, standard error, standardized estimates, and significance of the model’s regression weights (PUSAS Physical and Social Disorder); absolute and relative indexes assessing the goodness of fit.

		Estimate	S.E.	Standardized Estimate	*p-*value
ER OL ➔ PUSAS PSD 19		−0.334	0.123	−0.196	**0.007**
ER OL ➔ CAS		−0.010	0.040	−0.019	0.801
ER OR ➔ PUSAS PSD 19		−0.018	0.135	−0.01	0.893
ER OR ➔ CAS		0.046	0.044	0.077	0.295
ER OL ➔ DASS D		0.115	0.131	0.065	0.378
ER OL ➔ DASS A		0.099	0.114	0.064	0.385
ER OL ➔ DASS S		0.108	0.135	0.059	0.424
ER OR ➔ DASS D		−0.237	0.143	−0.122	0.097
ER OR ➔ DASS A		−0.099	0.125	−0.058	0.428
ER OR ➔ DASS S		−0.214	0.148	−0.106	0.147
PUSAS PSD 19 ➔ PUSAS PSD 22		0.395	0.060	0.413	**0.000**
CAS ➔ PUSAS PSD 22		0.207	0.241	0.070	0.391
DASS S ➔ PUSAS PSD 22		0.273	0.10	0.309	**0.006**
DASS A ➔ PUSAS PSD 22		0.012	0.119	0.011	0.920
DASS D ➔ PUSAS PSD 22		−0.059	0.108	−0.064	0.586
	CMIN/DF	RMSEA	NFI	RFI	CFI
Goodness of fit Indexes	1.837	0.067	0.985	0.914	0.963

Bold values are significant values.

**Figure 4:**
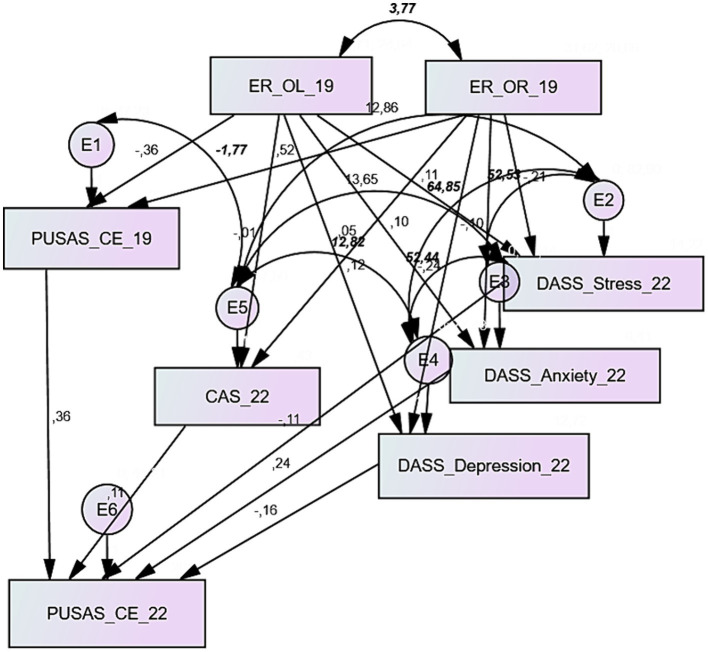
Path diagram of the model interpreting the PUSAS Collective Efficacy; parameters in regular text explain the standardized estimates of the regression weights, and parameters in bold italics explain covariances.

The path diagram concerning the PUSAS Collective Efficacy is portrayed in Figure 4. The analysis results provided excellent fitting (Table 5): the CMIN/DF ratio was below 2.0 (0.898), RMSEA was 0.000, NFI, RFI and CFI were above 0.95 (respectively 0.992, 0.957 and 1.000). As seen in the previous models, ego resiliency showed a significant effect on the PUSAS collective efficacy measured in 2019, but with both the dimensions of this construct, openness to life experience provided a negative regression weight (standardized γ: −0.364; *p* < 0.000) and optimal regulation a positive one (standardized γ: −0.524; p < 0.000). Again, the 2019 score in PUSAS collective efficacy was a significant predictor of its measure collected in 2022 (standardized γ: 0.356; *p* < 0.000), and the positive effect of the stress dimension of the DASS-21 on the collective efficacy score in 2022 (standardized γ: 0.244; *p* < 0.029). All the covariances were significant.

**Table 5:** Estimate, standard error, standardized estimates, and significance of the model’s regression weights (PUSAS Collective Efficacy); absolute and relative indexes assessing the goodness of fit.

		Estimate	S.E.	Standardized estimate	*p-*value
ER OL ➔ PUSAS CE 19		−0.364	0.102	−0.244	**0.000**
ER OL ➔ CAS		−0.010	0.041	−0.018	0.802
ER OR ➔ PUSAS CE 19		0.524	0.112	0.322	**0.000**
ER OR ➔ CAS		0.046	0.044	0.076	0.299
ER OL ➔ DASS D		0.115	0.131	0.065	0.378
ER OL ➔ DASS A		0.099	0.114	0.064	0.385
ER OL ➔ DASS S		0.108	0.135	0.059	0.424
ER OR ➔ DASS D		−0.237	0.143	−0.122	0.097
ER OR ➔ DASS A		−0.099	0.125	−0.058	0.428
ER OR ➔ DASS S		−0.214	0.148	−0.106	0.147
PUSAS CE 19 ➔ PUSAS CE 22		0.356	0.063	0.371	**0.000**
CAS ➔ PUSAS CE 22		0.111	0.222	0.043	0.618
DASS S ➔ PUSAS CE 22		−0.110	0.093	−0.142	0.237
DASS A ➔ PUSAS CE 22		0.244	0.112	0.265	**0.029**
DASS D ➔ PUSAS CE 22		−0.161	0.100	−0.201	0.108
	CMIN/DF	RMSEA	NFI	RFI	CFI
Goodness of fit indexes	0.898	0.000	0.992	0.957	1.000

Bold values are significant values.

**Figure 5:**
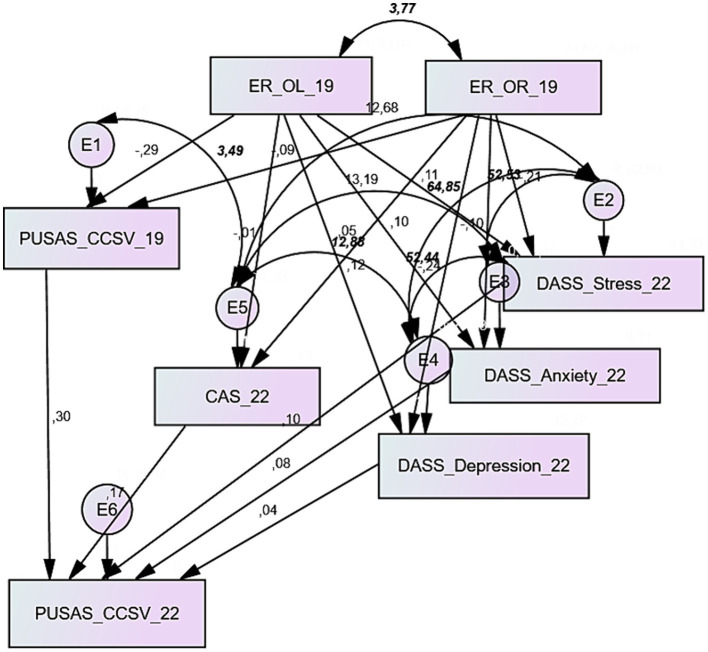
Path diagram of the model interpreting the PUSAS Concern about Crime and Sense of Vulnerability; parameters in regular text explain the standardized estimates of the regression weights, and parameters in bold italics explain covariances.

The last model analyzed in this research was related to the PUSAS concerns about crime and sense of vulnerability. The path diagram is represented in Figure 5, while Table 6 provides the regression weights estimate and the fit indexes. This model, too, was characterized by an excellent fit, with adequate absolute and relative fit indexes that respected the cut-off criteria. The CMIN/DF ratio was largely below 2.0 (1.096), the value of RMSEA was 0.023, and NFI, RFI, and CFI all presented measures above or equal to 0.95 (respectively 0.991, 0.948, 0.999). All the covariances were significant, but only two out of all the relationships in the model were detected as significant: the effect of ego resiliency’s openness to life experiences on the PUSAS concern about crime, with a negative regression weight (standardized γ: −0.289; *p* < 0.009), and the effect of PUSAS concern about crime assessed in 2019 with the 2022 measure, with a positive regression weight (standardized γ: −0.298; p < 0.000).

**Table 6:** Estimate, standard error, standardized estimates, and significance of the model’s regression weights (PUSAS Concern about Crime and Sense of Vulnerability); absolute and relative indexes assessing the goodness of fit.

		Estimate	S.E.	Standardized estimate	*p*-value
ER OL ➔ PUSAS CCSV 19		−0.289	0.110	−0.190	**0.009**
ER OL ➔ CAS		−0.010	0.040	−0.019	0.800
ER OR ➔ PUSAS CCSV 19		−0.092	0.120	−0.055	0.442
ER OR ➔ CAS		0.046	0.044	0.077	0.293
ER OL ➔ DASS D		0.115	0.131	0.065	0.378
ER OL ➔ DASS A		0.099	0.114	0.064	0.385
ER OL ➔ DASS S		0.108	0.135	0.059	0.424
ER OR ➔ DASS D		−0.237	0.143	−0.122	0.097
ER OR ➔ DASS A		−0.099	0.125	−0.058	0.428
ER OR ➔ DASS S		−0.214	0.148	−0.106	0.147
PUSAS CCSV 19 ➔ PUSAS CCSV 22		0.298	0.058	0.339	**0.000**
CAS ➔ PUSAS CCSV 22		0.175	0.208	0.071	0.400
DASS S ➔ PUSAS CCSV 22		0.099	0.085	0.136	0.243
DASS A ➔ PUSAS CCSV 22		0.076	0.101	0.089	0.449
DASS D ➔ PUSAS CCSV 22		0.042	0.091	0.056	0.647
	CMIN/DF	RMSEA	NFI	RFI	CFI
Goodness of fit indexes	1.096	0.023	0.991	0.948	0.999

Bold values are significant values.

## Discussion

After the pandemic’s beginning in 2019, many scholars devoted their research to debating the social and psychological consequences of the COVID-19 virus. Criminologists also analyzed the crime rates during this period, sometimes with heterogeneous evidence.

Scholars agreed on the decline of certain property crimes, like robbery, theft and burglary (Lopez and Rosenfeld, 2021; Perez-Vincent et al., 2021; Regalado et al., 2022), but the literature results showed heterogeneous results if violent crimes were considered: some authors founded not significant changes in violent behaviors (Lopez and Rosenfeld, 2021; Perez-Vincent et al., 2021), or a decline in homicides (Liu et al., 2022), other scholars provided evidence that during the pandemic this kind of crimes increased its rate (Meyer et al., 2022; Regalado et al., 2022). Domestic violence (Piquero et al., 2021; Kourti et al., 2023; McNeil et al., 2023) and cybercrime proliferated significantly during the pandemic (Kemp et al., 2021; Hoheisel et al., 2023).

This research proved that the feelings of safety enhanced during the coronavirus pandemic, but the roots of this phenomenon probably can be detected even before the spread of COVID-19. These findings may appear contradictory with the literature, with some criminologists suggesting that fear levels are relatively stable over time and do not really respond to fluctuations in crime rates (Warr, 1995; Ditton et al., 2000), due to the mismatches between the level of crime and individual-level fear rates. In contrast, more recent literature demonstrated that fear levels do follow crime trends (Smeets and Foekens, 2018), which is coherent with the results of this paper. The drop in feelings of unsafety was underlined by Pohl and Buil-Gil (2023), who analyzed the decrease in the worry about crime just before the pandemic, or Glas (2023), who operationalized unsafety with the fear of crime and linked the same trend to changes in the economic status and a true decrease in crime rates.

However, the findings provided by the present research also showed that the improvement in the feelings of safety was not generalized. A significant change was detected in the general measure of unsafety, in the score changes related to perceived physical and social disorder and concerns about crime and sense of vulnerability. Still, the perception of collective efficacy did not decrease significantly. One of the most important reasons for this lack of improvement in the quality of neighborhood social relationships must be searched in the effects of COVID-19 restrictions and distancing policies that affected the communities.

A significant body of literature in the last few years focused on the impact of the pandemic on the social sphere. A recent literature review (Alizadeh et al., 2023) reported dramatic psychological and emotional effects, exacerbation of segregation and poverty, disruption in educational systems and formation of an information gap, as well as declining trends of social capital among communities, all factors that can be tracked inside the social disorganization theory, one of the most important pillars explaining the unsafety or fear of crime.

Collective efficacy is perhaps that dimension of urban safety on which the pandemic has had the most significant impact. For example, it is the only dimension whose 2019 scores are not correlated to those of the other dimensions of the unsafety measured in 2022, while this has not been recorded for the evaluation of physical and social disorder or concern for crime. High levels of collective effectiveness seem to be associated with high performance in the optimal regulation of ego resilience in 2019, even if this association appears to be weaker in 2022, while a high collective efficacy, and only in 2019, is associated with low openness to life experiences scores. After the pandemic, low levels of collective efficacy were associated with depression but not with the specific measure of coronavirus anxiety. Even if not explicitly, the sum of these results lets us hypothesize the erosion of the pandemic on this aspect of the perception of safety.

Looking at the psychological dimensions considered in the study, the findings suggested the importance of resilience and other measures of general distress in modifying the levels of unsafety and fear of crime. However, they are not always consistent with the evidence in the literature.

Ego-resiliency is a personality trait that reflects “resourcefulness, sturdiness of character, and flexibility of functioning in response to varying environmental circumstances” (Luthar et al., 2000, p. 546).

Recently, some longitudinal research proposed a Trait Hypothesis of ER, which resulted in a stable, protective factor against stressful events and a predictor of positive affect irrespective of stressful events in a variety of social contexts (Vecchione et al., 2010; Kim et al., 2015; Karairmak and Figley, 2017; Liew et al., 2018). ER has been found to be associated with positive social relations, the use of social support networks, feelings of social support and cohesion in different community contexts (Taylor et al., 2014; Chang and Yarnal, 2016; Lozano et al., 2016; Cha and Lee, 2017).

Although ER has been considered a stable trait, more recent conceptualizations proposed that it may be viewed as a modifiable process, which may be improved through ER-oriented psycho-social interventions (Kalisch et al., 2015). A recent systematic review was conducted on 43 randomized controlled trials and identified several interventions to promote community resilience, including core ingredients, such as problem-solving, coping based on social support seeking, and mindfulness emotion regulation skills (Chmitorz et al., 2018).

The research findings described in this paper confirmed the positive impact of ego-resiliency on the unsafety dimensions. In particular, individuals oriented to optimal regulation have a higher collective efficacy and a lower concern about unsafety in general. In contrast, high scores in openness to life experiences are predictive of a lower perceived physical and social disorder, worry about crime and a lower general sense of unsafety. These significant associations were detected only with the data of the first wave of interviews (2019); they are inconsistent with the results of the second wave: the only significant correlation was found with collective efficacy, as previously commented. This lack of association with the unsafety measures collected after the pandemic was probably linked to the pandemic effect that modified the individual’s ego resiliency trait, even if scholars considered it a stable trait.

Several studies (Przepiórka et al., 2021; Goryczka et al., 2022) found a direct effect of ego resiliency on general distress, defined by depression, anxiety, and stress. The intensity of ego resilience correlated negatively with general distress and positively with the level of satisfaction with life. Ego-resiliency, also in these cases, seems to be a protective factor for the onset of psychopathology despite the severity of the pandemic situation. Ego-resilient individuals might have the ability to quickly and effectively restore their psychological balance after facing adversity. Moreover, optimistic thinking is a characteristic of ego-resilient individuals that helps to enhance positive emotions and ensures higher tolerance toward negative emotions, contrasting depression and anxiety. Many studies have explored the impact of ego resiliency on people’s functioning during the pandemic.

A study by Skalski et al. (2021) shows that ego-resiliency allowed for predicting the level of anxiety over COVID-19 and the adverse trauma effects. Furthermore, perceived social supports (in our case, comparable to collective efficacy) decrease stress and anxiety levels. The co-occurrence of personality traits (ego-resiliency) and environmental resources (social support) is essential for optimal adaptation to the traumatic event and maintaining mental health. Coronavirus anxiety mediated the relationship between mental resources and negative trauma effects. Covid anxiety may be a marker of mental functioning during the pandemic. Both ego-resiliency and perceived social support alleviate COVID anxiety and negative trauma effects.

Higher levels of ego-resiliency are correlated to lower levels of depression and stress, excluding anxiety (Kubo et al., 2021). Low ego-resiliency may adversely affect mental health due to limited daily activities outside the home due to fear of COVID-19 people.

The findings of this study suggested that ego-resiliency did not correlate with COVID-19 stressors and mental health during the pandemic. According to the authors, the absence of a correlation between ego-resiliency and anxiety and coronavirus anxiety indicated that during the pandemic, these emotions helped people adapt more effectively to this situation. In other words, coronavirus anxiety stimulated adaptation behaviors without the help of the resources provided by the resiliency trait. Stress and depression are counterproductive; ego-resiliency helps individuals to reduce these emotions, but coronavirus anxiety does not; therefore, ego-resiliency does not interfere with this.

A study by Dębski et al. (2021) shows a negative correlation between ego resiliency and the severity of anxiety during COVID-19. More specifically, openness to life experiences could be associated with perceived anxiety, while optimal regulation could play a role in its decline. Optimal regulation supports task-based dealing with crises, which at the same time should lower and inhibit the anxiety developed in situations of uncertainty related to the anticipation of the effects of a crisis. Openness to life experiences can be associated with the search for sensations, connected with the tendency to take risks, and thus with the possibility of feeling emotional tension.

In the study of Erden et al. (2023) severity of depression is not related to coronavirus anxiety, but anxiety is. This is not in line with studies that showed a positive correlation between depression and coronavirus anxiety. This finding may indicate that high levels of depression are associated with better-coping strategies for coronavirus anxiety, in fact, this sample uses active coping strategies such as “planning” (involves thinking and developing action strategies about how best to deal with the problem and what steps to take), that is negative relate to coronavirus anxiety.

In contrast with the literature, the results provided by this study showed a lack of association between ego resiliency and its subscales and the symptoms of general distress or coronavirus anxiety. It’s necessary to point out that there was a temporal gap of 3 years between the administration of the ego-resiliency measure (2019) and the scales administered in 2022 (CAS and DASS-21), a long period of dramatic experiences that probably modified the characteristics of individual’s ego resiliency trait.

Symptoms of general distress and coronavirus anxiety were strongly associated. However, consistent with the literature, the results of this study also proved that these measures are positively associated with the different dimensions of the perception of unsafety measured in 2022, except for collective efficacy, which, as commented previously, is negatively correlated only with depression. It is difficult to justify the findings related to the association between coronavirus anxiety, measured in 2022, and the perception of unsafety detected with the 2019 wave of interviews. Even in this case, the greater the unsafety, the greater the measurement obtained from the CAS, except again for collective efficacy, whose correlation was not significant. This issue is even more unexplained considering the symptoms of general distress measured in 2022, that are in no way associated with the measures of perceived unsafety collected at baseline.

The results of the structural equation models tested in this study, which analyzed the relationships mentioned above in terms of dependence between the variables in a network of relationships described by the theoretical model proposed in this research, offer an even more precise reading of the phenomenon. All models, analyzed according to the different dimensions of the PUSAS scale (physical and social disorder, collective efficacy, concern about crime and sense of vulnerability) and the overall score of the scale, share that coronavirus anxiety did not influence the measured unsafety after the pandemic. A general lack of effect of depression symptoms on unsafety was observed as well. The same conclusion concerns the lack of influence of ego-resiliency on coronavirus anxiety and general distress symptoms.

However, different pathways were observed before the pandemic about the effects of ego resiliency on the perceived unsafety. Optimal regulation was effective in contrasting a general sense of unsafety and enhancing collective efficacy, whilst openness to life experiences helped individuals reduce their worry about physical and social disorder and their concerns about crime and sense of vulnerability. But, individuals with an attitude to openness to life experiences suffered from a lower perception of collective efficacy.

Although general distress symptoms are significantly correlated with COVID-19 anxiety, only stress and anxiety measured by the DASS-21 appear to have an effect on unsafety measured in 2022, almost as if this is not the case. It is an effect of the pandemic, but rather an individual trait independent of the negative experiences experienced during COVID-19. Anxiety, in particular, was found to be a predictor of collective efficacy in a direct relationship.

A particular comment deserves the lack of effect of general distress symptoms on the dimension of unsafety regarding worry about crime and the sense of vulnerability. As highlighted earlier in the literature, the study of urban unsafety has traditionally been grounded on the concept of fear of crime, a construct for which researchers still struggle to identify a shared definition. This lack has produced the proliferation of instruments whose measurement validity has frequently been questioned, so much to undermine their specificity. Some authors (Ferretti et al., 2023) have highlighted that unsafety must probably be distinguished from fear of crime, so much so that it deserves specific measurement tools. If we bring this point of view to the extreme consequences, it could be reasonable to think that the studies cited previously on the association between symptoms of general distress and fear of crime could present the problem of a misrepresented construct. The data from the present study seem to confirm this statement: the study of structural models has shown that anxiety and stress can be predictors of unsafety in general, of the perception of physical and social disorder and collective efficacy, but not of the specific dimension of scale regarding concern about crime.

## Conclusion and future perspectives

The data from the study have allowed us to deepen our knowledge of the relationships between some psychological determinants and variations in the sense of unsafety, even if the role of pandemic events, such as the advent of COVID-19 between 2019 and 2022, is not particularly evident, a topic that deserves further research.

Individual characteristics determine feelings of safety or unsafety, not COVID-19’s impact. It is important to identify the most vulnerable individuals to plan targeted interventions. Rather than focusing on collective social measures, interventions to reduce feelings of unsafety should be directed toward individual clinical care. These interventions should take into account the impact of various factors on both the subjective quality of life and the perception of social context. Therefore, it is necessary to work on these factors at an individual level to improve the situation.

Among the limitations of this study that are worth mentioning is the lack of a baseline measure of general distress. However, as previously mentioned, these measures were not included in the first wave of interviews, given the impossibility of predicting the subsequent pandemic.

## Data availability statement

The raw data supporting the conclusions of this article will be made available by the authors, without undue reservation.

## Ethics statement

The studies involving humans were approved by the University of Siena CAREUS (Committee for the ethical research in human and social sciences) Careus Opinion N° 57/2022. The studies were conducted in accordance with the local legislation and institutional requirements. The participants provided their written informed consent to participate in this study.

## Author contributions

FF: Conceptualization, Data curation, Formal analysis, Investigation, Methodology, Supervision, Writing – original draft, Writing – review & editing. GG: Writing – original draft, Writing – review & editing. AM: Writing – original draft, Writing – review & editing. AU: Conceptualization, Data curation, Methodology, Writing – original draft, Writing – review & editing.
